# Building our research administrator workforce as our clinical and translational research programs become increasingly complex

**DOI:** 10.3389/fphar.2023.1295255

**Published:** 2023-10-24

**Authors:** Calvo Kayla, Phillips Jennifer, Burks Sandra, Karen C. Johnston

**Affiliations:** ^1^ Integrated Translational Health Research Institute of Virginia (iTHRIV), University of Virginia, Charlottesville, VA, United States; ^2^ Department of Neurology, University of Virginia School of Medicine, Charlottesville, VA, United States

**Keywords:** workforce development, professional development, research administration, mentoring, research team science

## Abstract

**Introduction:** Research administrators (RA’s) are critical members of the research workforce. For purposes of this article, research administrators are personnel who support the development, compliance, management, and financial oversight of sponsored research. There are currently very few institutional career development and mentoring programs available to research administrators. Recruitment and retention of quality research administrators has been especially challenging across the country in recent years.

**Methods:** In an effort to address this gap in training and to increase recruitment and retention, the integrated Translational Health Research Institute of Virginia (iTHRIV), a collaborative NIH-NCATS funded Clinical Translational Science Award (CTSA) hub, has developed an innovative program of workforce development and mentoring for research administrators. This article provides an overview of one institutional training and development initiative, the Research Administration Program for Training and Resources (RAPTR). RAPTR provides training, resources and mentoring to develop a Community of Practice.

**Results:** The program provides a forum where research administrators can share ideas, practices, and challenges.

**Discussion:** This manuscript describes the benefits and lessons learned from our early experience in this program. We highlight selected components that may be generalizable to other institutions and describe individualized components, which require local policies and processes.

## Introduction

Research administrators provide specialized and unique skills that are integral to the success of a research team. Recruitment and retention of talented research administrators has been especially challenging across the country in recent years. The increase in remote work positions has allowed research administrators to choose the most desirable positions in which to work. In our experience, research administrators are seeking the most competitive salaried positions with institutions that provide flexibility in their day-to-day work life balance, a collaborative work environment, and offers desirable career development opportunities.

It is well described that employee satisfaction improves when career development opportunities are available and such programs likely improve recruitment and retention (Wau and Purwanto, 2021). Employee retention and engagement is critical for organizations because employees are the driving force to achieve the development and accomplishment of the organization’s goals and objectives (Aguenza and Som, 2012). Many studies suggest that organizations with greater personnel stability perform better than those with less stability (Pitts et al., 2011). Additionally, there is a danger of a loss of institutional memory in organizations with high levels of turnover amongst their professional staff (Shaw et al., 2005; Mustapha et al., 2011). The biggest factor in attracting, and most importantly, retaining key employees is culture (Aguenza and Som, 2012). Employees need to feel that they are part of a team, connected to the vision and direction of the organization (Ryan Carruthers, 2023). We believe a focused, connected, and collaborative team leads to greater stability in the workforce resulting in greater discovery and impact. Formal training and career development programs may help create and sustain this workforce.

Research administration is often described as a “found career.” (Srainternational, 2023) These positions almost exclusively exist in research institutes and academic research institutions. Due to the exclusivity of these positions, there are very few options available for formal education. The few existing commercially available formal training programs are often expensive and overly generalized in subject matter. Professional organizations such as the National Council of University Administrators (www.ncura.edu) and the Society of Research Administrators International (www.stainternaltional.org) provide some opportunities for training, professional development, and networking, however it does not meet all needs. This leaves individual institutions with the responsibility to train their own research administrators. Even with prior experience, all research administrators entering new positions require training in specific institutional policies and processes. Those who work in a decentralized system may work in isolation, and may find it challenging to acquire this institution-specific knowledge without a structured program. Managers and supervisors may be unable to adequately train and provide support for these highly specialized roles. These factors may result in feelings of isolation, poor job satisfaction, and low retention rates without additional career development programs.

Mentoring programs can contribute to job satisfaction and career growth. Such programs have been successful in academic research for career development of other team members (Sambunjak et al., 2006) but have not been well described for research administrators. Researchers often utilize mentorship to grow and develop in their chosen field with mentors sharing their knowledge, experience, and skills. iTHRIV recognized the importance of the mentorship model as a critical component to support career growth and avoid isolation for our research administrators. Once a community of mentors and mentees is created, these programs can leverage the benefits of a Community of Practice.

A Community of Practice is a group of people who share a concern or a passion for something they do and learn how to do it better as they interact regularly (Etienne and Beverly Wenger-Trayner, 2022). Members of a Community of Practice are practitioners who develop a shared repertoire of resources: experiences, stories, tools, ways of addressing recurring problems—in short, a shared practice (Etienne and Beverly Wenger-Trayner, 2022). Communities of Practice can help with problem solving, developing confidence, sharing of resources, mapping knowledge, and identifying gaps (AMI Communities of Practice, 2023). iTHRIV sought to leverage the concepts of a Community of Practice in the development of RAPTR.

Formal training and career development programs to nurture the research administrator workforce in academic institutions are not well described in the literature. In addition, though mentoring programs are ubiquitous for the research workforce, they are conspicuously missing for research administrators at many institutions. In an effort to address the gap in professional development including formal training, mentorship, and need for a Community of Practice, iTHRIV developed this innovative program, which includes a workforce development series, peer mentoring, and office hours for group problem solving specifically for research administrators. RAPTR is intended to facilitate growth opportunities at all levels and provides a supportive environment with expert peers that is intended to improve recruitment and retention of these important research team members.

## Methods

RAPTR was developed in stages to provide both virtual and in-person training, expert guidance, and written resources for participants. The program has 3 major components: 1) online training content; 2) a paired mentoring program; and 3) a Community of Practice with facilitated office hours. Nine invested members of the research administration community at the University of Virginia (UVA) initiated the RAPTR Steering Committee in late 2019. The initial charge of the committee was to assess training needs for the workforce, prioritize, and develop necessary materials/programs. Basic descriptive statistics were used for program evaluation. The committee regularly reviews feedback on all components and makes decisions about the continuation of current programing and the development of new resources.

### Online resources

**FIGURE 1:**
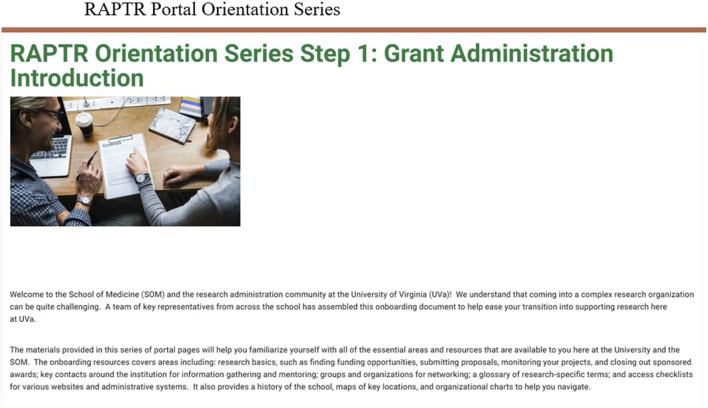
RAPTR portal orientation series.

**FIGURE 2:**
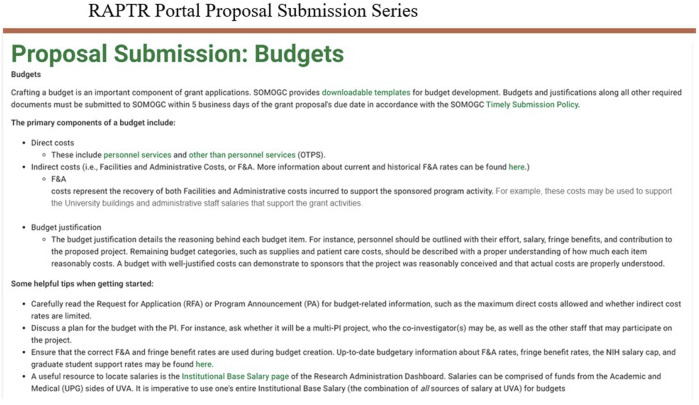
RAPTR portal proposal submission series.

The Steering Committee prioritized the order of which new materials would be developed. The Orientation Series was developed first. This series (Figure 1) provides a new research administrator with a list of important contacts, information on system access and training, policies and guides, opportunities for professional development, and a glossary of terms. A second series focuses on the Proposal Submission process and includes key definitions, links to budget templates and institutional budget information (Figure 2), and tips for managing internal submission processes with a focus on NIH submissions. Both series are offered in a centralized online platform, the iTHRIV Research Concierge Portal (Portal) (Loomba et al., 2022).

### Mentoring program (RAMP UP)

Following the launch of RAPTR and based on feedback, it became apparent to the Steering Committee that a formal structured mentoring program would be advantageous for the RA workforce. This led to the development of the iTHRIV Research Administration Mentoring Program and University Partnership (RAMP UP). This initial pilot program within RAPTR creates a paired mentor/mentee structure to support the individualized growth and success of the participants. The pilot program solicited applications from research administrators primarily from the UVA School of Medicine, for both mentees and mentors though additional applications from the School of Engineering and Applied Sciences and UVA’s central Office of Sponsored Programs (OSP) were also accepted. The School of Medicine was selected for the pilot as it has research administration community who expressed a need for the program. The application for mentors and mentees was created using REDCap (Harris et al., 2009; Harris et al., 2019). Mentors were required to have at least 5 years of experience in the field of research administration. The application included a section that listed subject areas in research administration and asked the applicants to note their experience/expertise. Applicants were also asked to describe their interest in and philosophy for mentoring. Supervisors were required to approve participation and final selection of mentors followed interviews by Steering Committee members.

The Steering Committee matched mentors with mentees considering mentor experience and mentee desired growth. Once selected, new mentors were required to attend a 4-h mentor training workshop hosted by a Center for Improving Mentored Experiences in Research (CIMER) trained facilitator which covered major mentoring concepts (Table 1). (Pfund et al., 2013) Though the initial cohort included thirteen mentor/mentee dyads from across UVA, RAMP UP encouraged mentees to connect with other mentors in the cohort for additional expertise and/or support. Dyads in the first-year pilot met regularly and the entire cohort met once a month for a workshop, feedback session, and/or cohort office hours. These group sessions created a small Community of Practice. Additionally, the group sessions allowed early career research administrators to appreciate their potential as mentors especially to those new to the institution. This structure provides a sustainability plan in that mentees are encouraged to advance to mentor status when appropriate thus creating a pathway for career development and a growing pool of mentors.

**TABLE 1:** RAMP UP mentor training workshop topics.

Communication Strategies
Aligning Expectations
Enhancing Understanding
Reflecting on Diversity
Fostering Wellbeing

### Early development of a community of practice

Recognizing the need to grow relationships and build community across a disconnected workforce, RAPTR began hosting virtual facilitated office hours to serve the School of Medicine RA community. Twice-monthly facilitated sessions encourage questions, discussion, and the sharing of best practices across the group. Leaders from the School of Medicine Office of Grants and Contracts and other members of the RAPTR Steering Committee moderate office hours. The RA community guides topics for discussion and often include budget development, proposal submission, institutional systems, and post award administration. Recent verbal feedback from participants requested that office hours move to a more structured format. RAPTR Office Hours were recently restructured to include the entire UVA research administration community. Each session begins with focused topic discussion led by subject matter experts and ends with open forum question and answer time.

Evaluation plans initially focused on utilization and informal feedback on programs but also includes formal evaluation of RAMP UP. The RAMP UP evaluation plan included an anonymous voluntary interim and end-of-program feedback survey. An annual follow-up survey will be distributed to review retention and career advancement rates. The Steering Committee regularly gathers all formal and informal feedback for discussion to inform continuous quality improvement efforts.

## Results

The RAPTR Orientation series publicly launched in the iTHRIV Portal in the spring of 2021. The Proposal Submission series followed in late 2021. Early results show consistent utilization of all RAPTR online Portal resources. According to Google analytics from June 2023 (Table 2), the web-based resources infer that users are returning to resources multiple times. For instance, the “Orientation: Required Systems and Training” Portal page has been viewed 159 times by 63 unique users (44% of the UVA research administration workforce). We continue to capture informal feedback on the usability and value of content from these offerings.

**TABLE 2:** Google analytics for RAPTR pageviews.

Resource title	User Type^0^	Pageviews*	Unique Pageviews^
Orientation (1): Grant Administration Introduction	New Users	125	73
	All Users	308	193
Orientation(3):Grant Administration Required Systems and Training	New Users	63	45
	All Users	159	118
Orientation(2):Grant Administrators Impo1iant Contacts	New Users	52	35
	All Users	130	91
Orientation (4):Grant Administration Inte1nal and Extemal Resom·ces	New Users	43	34
	All Users	104	79
Proposal Submission: Intemal F01ms and Routing	New Users	27	24
	All Users	72	63
School of Medicine Research Administration Po1tal for Training and Resources - RAPTR (START HERE !)	New Users	40	23
	All Users	162	109
Orientation(8):Grant Administration Professional Development	New Users	27	22
	All Users	74	56
Proposal Submission: Introduction	New Users	23	20
	All Users	68	60
Proposal Submission: Budgets	New Users	25	20
	All Users	74	61
Orientation(7):Grant Administration UVA Research Administration Policies	New Users	25	19
	All Users	69	55
Orientation (6):Grant Administration Listservs for Research Administrators	New Users	18	13
	All Users	63	40
Orientation(5):Grant Administration Institutional Research Administration Meetings	New Users	17	12
	All Users	62	43
Proposal Submission: Introduction	New Users	12	11
	All Users	46	39
Proposal Submission: Non-Federal Proposals	New Users	14	9
	All Users	36	26
Proposal Submission: Indirect Costs (F&A)	New Users	10	9
	All Users	40	29

**FIGURE 3:**
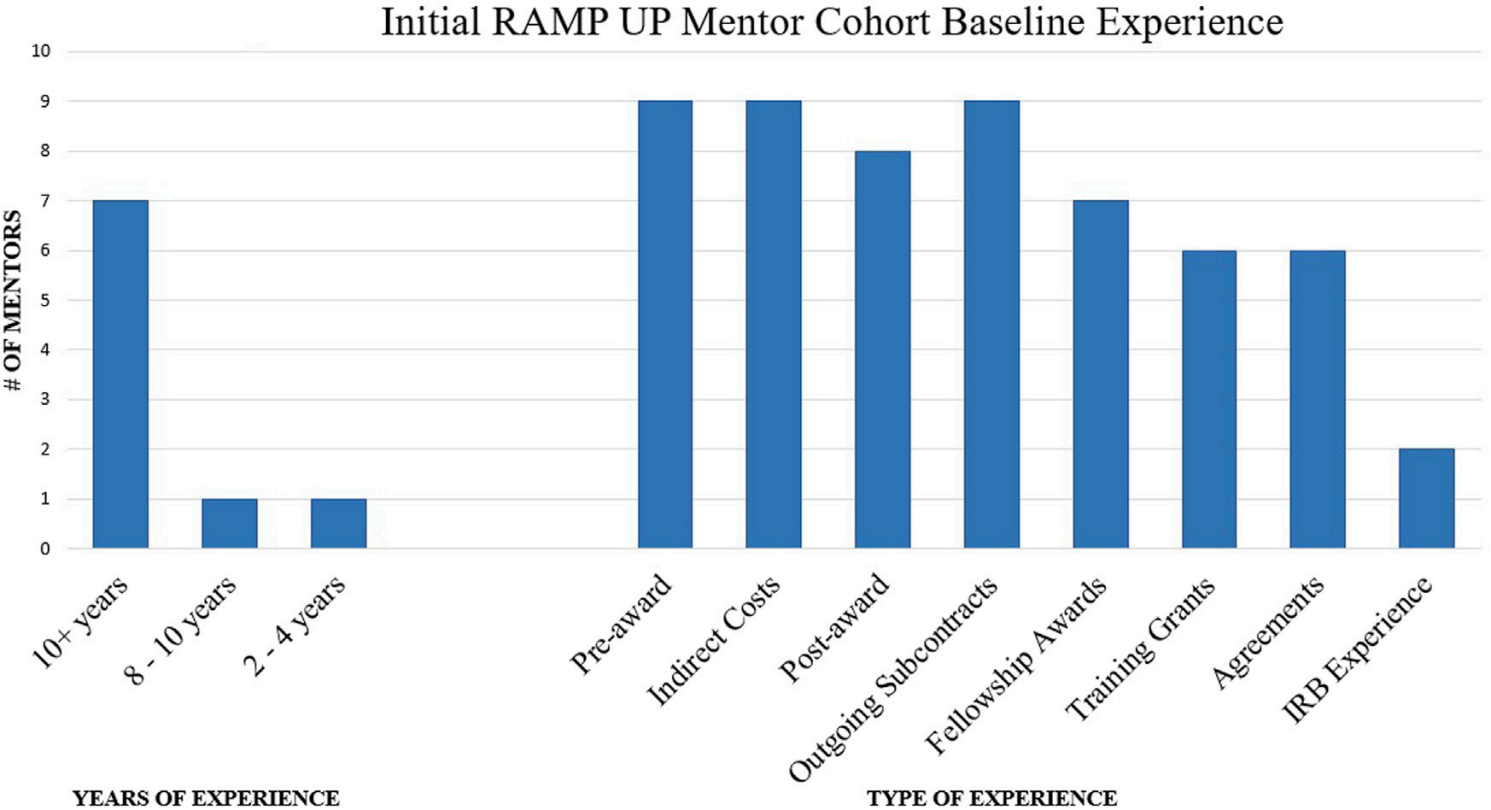
Initial RAMP UP mentor cohort baseline experience.

The RAMP UP Request for Mentor applications was released in June of 2022. Nine research administrators from two schools (School of Medicine and School of Engineering and Applied Sciences) and the central Office for Sponsored Programs applied to be mentors and demonstrated broad expertise and experience (Figure 3). Additionally four Steering Committee members served as mentors to complete the cohort.

**FIGURE 4:**
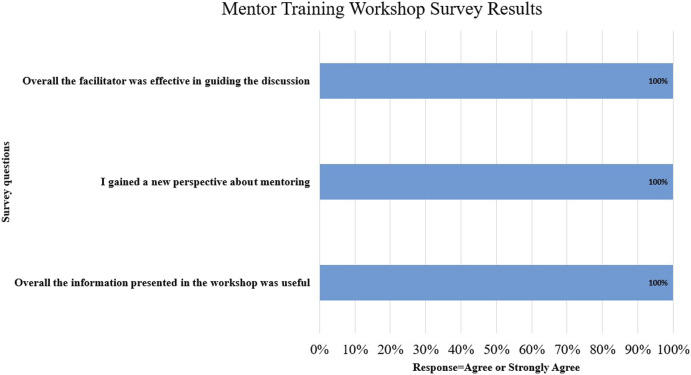
Mentor training workshop survey results.

All mentors attended the required mentor training workshop either in person or virtually. Post monthly session evaluations demonstrated strong support for the training session with a 44% response rate (Figure 4). Written feedback from the mentors included appreciation for both the large and small group activities as well as the section on effective communication.

**FIGURE 5:**
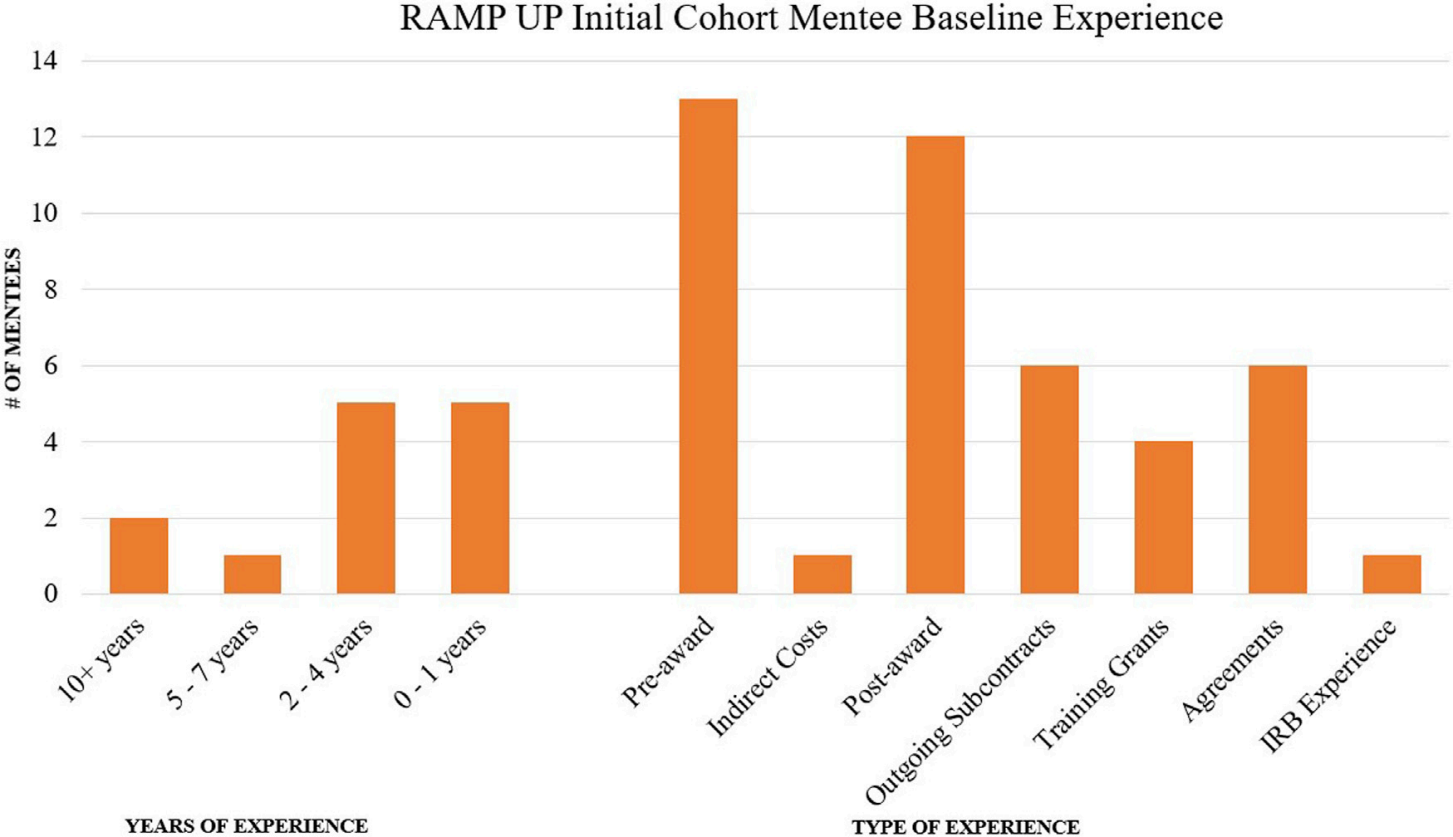
RAMP UP initial cohort mentee baseline experience.

The RAMP UP Request for Mentee applications was released in July 2022. Applications (*N* = 13) demonstrated variability in years of experience and expertise in the field (Figure 5). Applicants described several reasons for wanting to participate in the program including expanding knowledge of research administration, professional development, networking, and improving knowledge of the UVA system.

Two Steering Committee members interviewed each mentee and then the full committee determined the pairings with mentors. Each mentor was assigned a single mentee. An interim participation survey was distributed to the mentees in January 2023 and 10 mentees responded (77%). Ninety percent of the mentees stated that they were meeting regularly with their mentors and that the program was meeting their expectations. The mentees described the value and impact of having someone answer their questions, understand the challenges they were facing were not unique, and generally expressed gratitude for inclusion in the program. Feedback also included suggestions to have a more detailed syllabus and guide for mentors and mentees to follow, as well as creating a boot camp/training program specifically for new research administrators.

**FIGURE 6:**
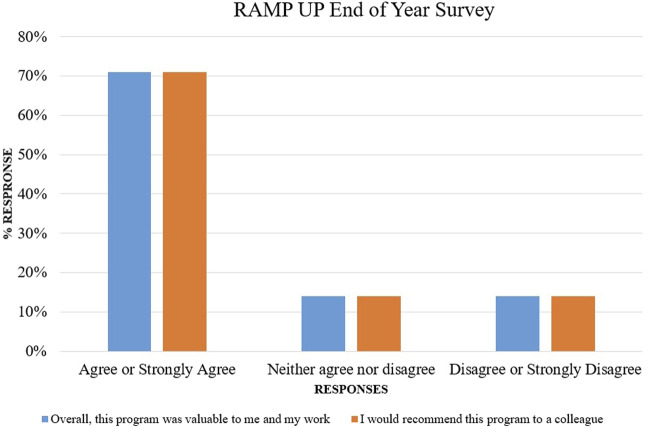
RAMP UP end of year survey.

The Steering Committee conducted an end of year survey in August 2023. All mentors and mentees were surveyed with a 39% response rate. The overall response to the program was positive with feedback similar to the interim survey (Figure 6). Other feedback demonstrated a need for additional tools, more structure, and support for the mentors. Clearly defined expectations were felt to be missing and will be addressed in future programs.

The facilitated office hours has an average of 10–15 attendees for each session. Early feedback showed that attendees appreciate the opportunity to informally ask questions and get answers from both peers and Leaders within the SOM Office of Grants and Contracts. Informal feedback and requests for specific content for office hours guides priority topics for upcoming sessions. As this is a new program, end of year feedback has not yet been solicited. Early markers for success for the newly structured office hours are positive.

## Discussion

RAPTR, which includes online training resources, a mentoring program, and facilitated office hours, has created an early Community of Practice of research administrators at UVA. This program was created to fill a gap in supporting a critical part of the research workforce. We believe that this will be an important recruitment tool as it provides both training and career development opportunities for new recruits. Introductory resources were created to provide a guide for new research administrators as they navigate a complex web of sponsors, grant proposals, post award management, and contracts. As our institution’s portfolio continues to grow and evolve and as awards become more complex, our program can be flexible by adapting to the needs of the workforce in all three areas (online resources, mentoring, facilitated office hours). Early results suggest that such a program can help equip research administrators with the tools and support they need to manage a growing research portfolio. Though specific program and process content may vary across institutions, programs like RAPTR may have generalizable professional development concepts that can help grow the research administrator workforce across the nation.

Evaluation of RAPTR is limited by a lack of long-term impact data. As the program continues to evolve and both formal and informal feedback is collected, we will more clearly be able to define the most impactful components. Future directions of our program include the formation of sub-committees under the guidance of the RAPTR Steering Committee to address individual components of the program. An education subcommittee will be charged with a re-design and dissemination of the currently available online resources to address recent institution wide financial and grant management systems changes affecting our entire research administration community. The continued need for these online materials is evident as the field and the systems advance rapidly. Additional resources will also be created to include topics relevant to the entire RA workforce at UVA. Facilitated office hours will be more topic guided (reporting, budgeting, payroll allocations, etc.) and will be made available to all RAs across the institution. Additional programs may include a boot camp for new research administrators and a foundational Research Administration Certificate program.

The RAMP UP sub-committee is evaluating the interim and year-end feedback and will offer a more structured mentoring program focused on professional development. In response to feedback, the program will provide a written guide for both mentors and mentees and develop a template for a compact between dyad members to align expectations. New workshop session topics for dyads will include Leadership, Building a Network, and Developing an Engaging Presentation. The inclusion of a capstone project that benefits the research administration community at UVA will be piloted as part of the 1-year mentoring program.

## Conclusion

**FIGURE 7:**
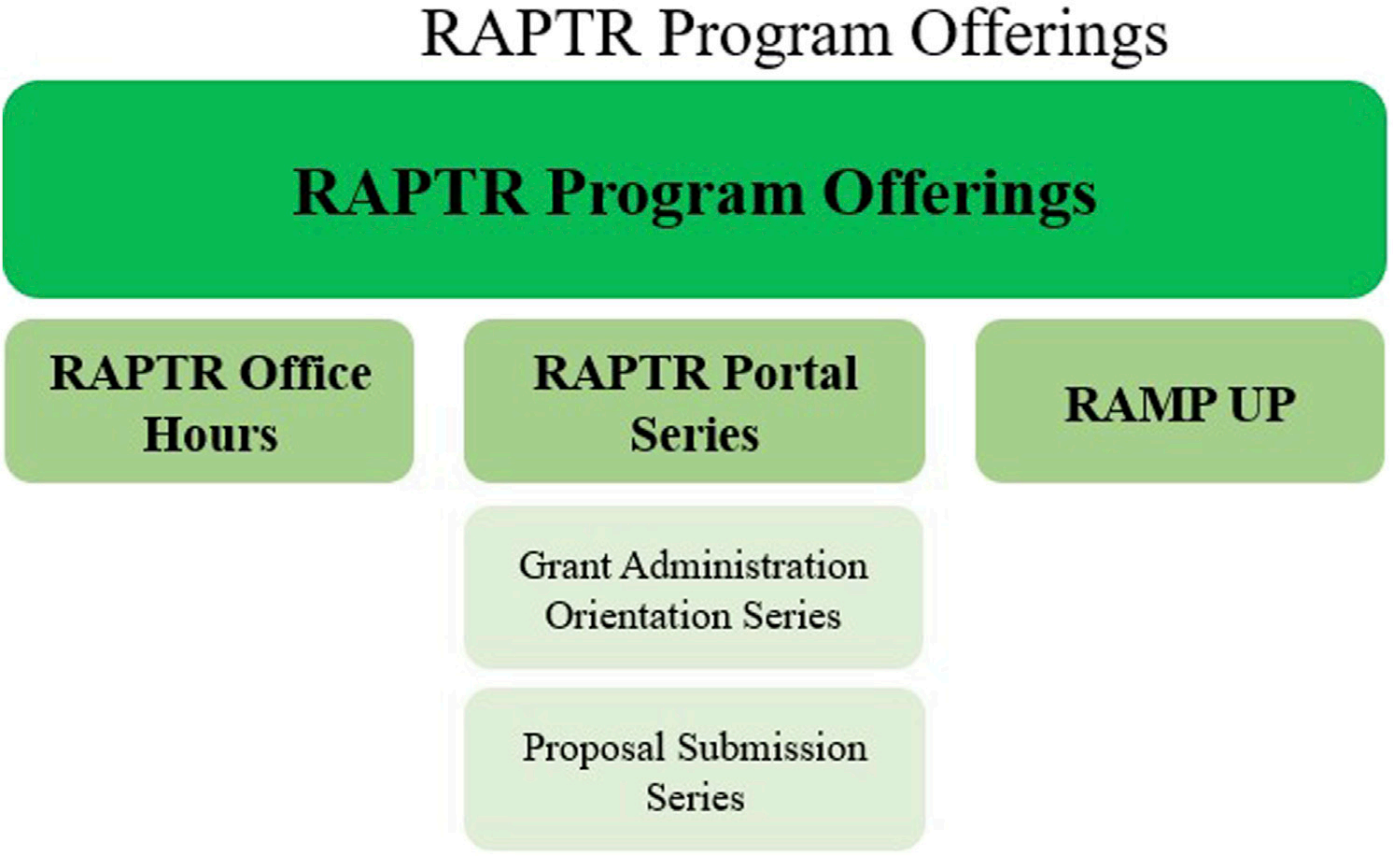
RAPTR program offerings.

Through the development of RAPTR, we are building a community of educators and learners with a passion and dedication for research administration. These programs (Figure 7) increase the knowledge, skills, and abilities and hopefully job satisfaction of an important part of the research workforce. This grass roots effort started in the UVA School of Medicine and is now engaging other schools, departments, and leadership within the institution.

RAPTR is just one approach to supporting the career development of our research administrators as part of our workforce development programs. Additional consideration of innovative and impactful approaches to grow this critically important component of our research teams is warranted.

## Data Availability

Publicly available datasets were analyzed in this study. This data can be found here: https://www.ithriv.org/raptr.
